# Predictive Factors for the Discontinuation of Pressurized Intraperitoneal Aerosol Chemotherapy: Enhancing Patient Selection to Improve Oncological Outcomes—A Single-Center Experience

**DOI:** 10.3390/cancers17020265

**Published:** 2025-01-15

**Authors:** Matteo Aulicino, Francesco Santullo, Cecilia Orsini, Luca D’Agostino, Martin Hübner, Hugo Texeira-Farinha, Manuela Robella, Olivia Sgarbura, Agustìn Bianco, Almog Ben-Yaacov, Federica Ferracci, Giorgio D’Annibale, Fabio Pacelli, Andrea Di Giorgio

**Affiliations:** 1 General Surgery Department, Università Cattolica del Sacro Cuore, 00168 Rome, Italylucadago93@gmail.com (L.D.); giorgio.dannibale01@icatt.it (G.D.); 2 Surgical Unit of Peritoneum and Retroperitoneum Surgery, Fondazione Policlinico Universitario Agostino Gemelli IRCCS, 00168 Rome, Italyandrea.digiorgio@policlinicogemelli.it (A.D.G.); 3 Service de Chirurgie Viscérale, Centre Hospitalier Universitaire de Lausanne (CHUV), University of Lausanne (UNIL), 1015 Lausanne, Switzerland; martin.hubner@chuv.ch (M.H.);; 4 Department of Surgical Oncology, Candiolo Institute for Cancer Research and Treatment, 10060 Torino, Italy; manuela.robella@ircc.it; 5 Department of Surgical Oncology, Cancer Institute Montpellier (ICM), University of Montpellier, 34298 Montpellier, France; 6 Department of Surgery, Chaim Sheba Hospital Medical Center, Tel-Hashomer, Ramat Gan 52621, Israel

**Keywords:** surgical oncology, PIPAC, discontinuation, peritoneal carcinomatosis, standard treatment, predictive factors

## Abstract

**Background**: Since 2011, Pressurized Intraperitoneal Aerosol Chemotherapy (PIPAC) has emerged as a promising treatment option for patients with peritoneal surface malignancies (PSM) who are not eligible for cytoreductive surgery (CRS). Repeated minimal-invasive treatment is one of the key features and the current empirical standard treatment (ST) consists of at least three administrations over about three months. However, many patients are unable to complete the full course, limiting the potential benefits of PIPAC. **Method**: This retrospective, single-center study assessed the completion rate of ST and identified the main causes and predictive factors for discontinuation. This study also evaluated the feasibility, safety, and efficacy of PIPAC and investigated whether improved patient selection over the years has resulted in better oncological outcomes. **Result**: Data from 168 patients treated with PIPAC between January 2017 and March 2023 for a total of 336 procedures showed that only 29% completed ST. Multivariate analysis identified ascites >500 mL and a prior history of bowel obstruction as significant predictors of discontinuation. **Conclusions**: Patients with radiological or clinical signs of obstruction should not be considered for PIPAC treatment, and ascites increases the risk of incomplete treatment. Larger studies are eagerly awaited to corroborate these findings and refine the selection criteria by disease entity.

## Simple Summary

Pressurized Intraperitoneal Aerosol Chemotherapy (PIPAC) is a viable treatment for patients with peritoneal surface malignancies (PSM) who are not eligible for cytoreductive surgery (CRS). The literature suggests that a minimum of three PIPAC procedures (empirical standard treatment) should be administered to optimize oncological outcomes. However, many patients are not able to complete three treatments for various reasons. The aim of this retrospective study was to identify causes and predictive factors for incomplete PIPAC treatment and, hence, to improve patient selection.

## 1. Introduction

Peritoneal surface malignancies (PSMs) are a heterogeneous group of tumors that differ in incidence, sensitivity to systemic therapies, and prognosis. Compared to other metastatic sites, systemic chemotherapy is less effective for peritoneal metastases (PMs). This might have to do with different molecular subtypes and tumor biology. Furthermore, reduced blood supply to the peritoneum, high interstitial pressure, and the peritoneum–plasma barrier are important pharmacokinetic limitations preventing systemic treatments from reaching and targeting tumor cells. Therefore, in recent decades, new techniques for delivering chemotherapy have been developed to increase local control of peritoneal disease, with promising results [1,2].

Nowadays, cytoreductive surgery (CRS), mostly in combination with consecutive hyperthermic intraperitoneal chemotherapy (HIPEC) [3,4,5], appears to be the only therapeutic option with curative potential in selected patients with a low peritoneal disease burden [6,7].

Since 2011, Pressurized Intraperitoneal Aerosol Chemotherapy (PIPAC) has been used as a treatment option for patients with PSM not eligible for CRS and HIPEC. This new technique increases drug concentrations in targeted peritoneal tissues while maintaining low plasma concentrations, thus limiting systemic toxicity [8,9,10]. Moreover, the concurrent performance of a diagnostic laparoscopy with peritoneal biopsies allows for close monitoring of the treatment response. The literature has demonstrated that encouraging oncological outcomes can be achieved with repeated PIPAC treatment [11,12,13]. However, many patients do not complete the standard treatment, thus probably limiting the benefits. The main aim of this study is to identify the rate of patients who received at least three PIPACs and to identify the most frequent causes and risk factors for PIPAC discontinuation. The secondary aim was to assess the feasibility, safety, and efficacy of PIPAC in terms of survival and regression of symptoms. Finally, we evaluated whether the progressive improvement in and increased attention to patient selection for PIPAC over the years has led to improved outcomes.

## 2. Materials and Methods

This is a retrospective single-center study based on a prospectively maintained database, which included patients undergoing PIPAC from January 2017 to March 2023 at the Department of Peritoneal and Retroperitoneal Surgery, Fondazione Policlinico Universitario Agostino Gemelli, IRCCS. Indication for PIPAC treatment was provided by a multidisciplinary team.

The inclusion criteria were age > 18 years, presence of primary peritoneal tumors (mesothelioma), or peritoneal carcinomatosis from a different primary tumor (colorectal, stomach, ovarian, pancreatic, hepatobiliary, appendix, breast, small intestine, and lung) not susceptible to CRS. The exclusion criteria were patients’ refusal, presence of retroperitoneal disease and/or other significant distant metastases at the time of the first PIPAC, presence of other concurrent cancer, a life expectancy of less than 3 months, inability to orally feed or the need for parenteral nutrition, and an Eastern Cooperative Oncology Group (ECOG) Performance Status > 2.

### 2.1. Outcomes Measures

Standard treatment (ST) was defined as patients receiving ≥ 3 PIPACs and was compared to patients who underwent only 1 or 2 PIPACs. The factors analyzed as predictors of PIPAC discontinuation included patient characteristics (age, gender, body mass index (BMI), ECOG [14], tumor characteristics (site of the primary tumor, synchronous/metachronous carcinomatosis, and Peritoneal Cancer Index (PCI) according to the Sugarbaker score [15]), symptoms before first PIPAC and after third PIPAC (ascites volume (ascites volume was measured at PIPAC1, based on the intraoperative aspirates), pain, nausea, dysphagia, and intestinal obstruction symptoms), and systemic chemotherapy treatment (number of chemotherapy lines, number of chemotherapy cycles, and bimodal treatment (bimodal treatment was defined as the patient being under systemic chemotherapy in the interval between PIPACs)). Reasons for PIPAC treatment discontinuation were also evaluated: disease progression (DP), patient’s wish, other medical reasons, conversion to curative CRS, impossible access to the abdominal cavity (non-access), death, negative histology, and PIPAC complications.

The secondary outcome was the evaluation of the efficacy of PIPAC treatment. Median overall survival (mOS) from PIPAC1 was compared between patients undergoing ST and those undergoing only 1 or 2 PIPAC procedures. Furthermore, the symptoms reported at PIPAC1 were compared with those after the completion of ST to assess the impact of PIPAC on symptoms.

To estimate the feasibility and safety of PIPAC, we evaluated the incidence of complications according to the CTCAEv5 classification [16] and 30-day and 90-day mortality rates among all PIPAC procedures.

Finally, we evaluated the evolution of oncological outcomes throughout our experience. To achieve this, we analyzed the annual differences in the percentage of patients who completed the standard treatment (ST) and compared the median overall survival of patients treated with PIPAC between January 2017 and December 2020 with those treated between January 2021 and March 2023.

### 2.2. Surgical Technique

The procedure was performed according to current recommendations and safety protocols [17,18,19].

Under general anesthesia, a 10–12 mm laparoscopic balloon trocar was placed in accordance with the open technique, and a capnoperitoneum of 12 mmHg at 37 °C was applied. Then, one or two 5 mm laparoscopic balloon trocars were placed. During the procedure, ascites was documented and eventually removed (with a sample sent for cytological examination), and a thorough laparoscopic exploration was performed to calculate the PCI and assess the extension of peritoneal disease. Finally, multiple biopsies were performed in different abdominal quadrants. The PIPAC treatment involved the delivery of a pressurized aerosol using a CAPNOPEN© nebulizer connected to a high-pressure injector, which was inserted into the 10–12 mm trocar with an inclination of 45° relative to the underlying peritoneum to optimize the diffusion of the chemotherapy within the abdominal cavity. The system was then kept in a steady state for 30 min (application time), and any remaining toxic aerosol was exhausted in a closed surgical smoke evacuation system to prevent its diffusion outside the abdominal cavity. Finally, the trocars were removed, and laparoscopy was completed without the need for abdominal drainage placement.

The combinations of chemotherapy used in our study were consistent with those reported in the literature: Oxaliplatin 92 mg/m^2^ was used for PM from colorectal and appendiceal carcinoma and for pseudomyxoma. The combination of Cisplatin 7.5 mg/m^2^ + Doxorubicin 1.5 mg/m^2^ was used for gastric, ovarian, pancreatic, and biliary tract peritoneal metastases, and mesothelioma. Since 2018, after the phase I study by Tempfer et al. [9], a higher dose of Cisplatin and Doxorubicin (10.5–2.1 mg/m^2^) has been adopted. In addition, in patients with peritoneal carcinomatosis from pancreatic carcinoma, the use of Nab-Paclitaxel 112.5 mg/m^2^ has been adopted since March 2022 within the controlled phase II Nab-PIPAC trial to evaluate the effectiveness of this drug [12,20].

Patients were scheduled for three PIPACs at 4–6-week intervals. In patients with bimodal treatment, the predefined washout period from systemic chemotherapy to PIPAC was 2 weeks, and 1 week from PIPAC to systemic chemotherapy.

Additional procedures were performed based on the surgeon’s evaluation. Bowel resections were performed in the case of isolated sub-obstructive peritoneal lesions. Adnexectomy was performed in patients where ovarian lesions were the only site of progression or the lesions were symptomatic [21].

### 2.3. Postoperative Treatment and Follow-Up

Adverse events were recorded either during the hospitalization period or reported by patients during outpatient visits. Between each PIPAC administration, patients underwent routine follow-up assessments, including hematological, radiological, and oncological marker measurements.

These results were reviewed by a multidisciplinary team, and additional PIPAC treatments were administered only after confirming the absence of disease progression.

### 2.4. Statistical Analysis

Continuous variables are reported as mean (Standard Deviation—SD) or median (Minimum–Maximum). Categorical variables are reported as numbers and frequencies (%). Multivariable analyses were performed using multinomial logistic regression integrating with univariate *p*-values ≤ 0.1. The statistical power of the multivariate analysis was calculated by setting the effect size f^2^ to 0.15 and the alpha error probability (α) to 0.05. Kaplan–Meier curves were used for graphical representation of the results. Statistical significance was considered for *p*-values < 0.05, and confidence intervals (CIs) were established at 95%. Statistical analysis and graph processing were performed using GraphPad Prism 9, the IBM SPSS software v29.0.1.0 platform, Excel v16.54, and G*Power 3.1.

## 3. Results

A total of 336 PIPAC procedures were performed on 168 patients with PM. Disease entities were gastric cancer in 69 patients (41%), pancreatic cancer in 46 (27.5%), colorectal cancer in 30 (18%), hepatobiliary cancer in 12 (7%), and other primary sites (ovarian, breast, lung, small bowel, and mesothelioma) in 11 (6.5%). The reasons for PIPAC discontinuation are summarized in Table 1.

Presence of abdominal pain (OR = 0.469, 95% CI [0.230–0.956], *p* = 0.037) and bowel obstruction symptoms before PIPAC1 (OR = 0.249, 95% CI [0.072–0.862], *p* = 0.028) and ascites > 500 mL during the first treatment (OR = 0.366, 95% CI [0.157–0.853], *p* = 0.02) were demonstrated to be predictors of standard treatment discontinuation. No other significant predictors of treatment discontinuation were identified among the evaluated factors (Table 2).

Prior history of bowel obstruction symptoms (OR = 0.264, 95% CI [0.74–0.947], *p* = 0.041) and ascites > 500 mL (OR = 0.375, 95% CI [0.157–0.900], *p* = 0.028) were found to be independent predictive factors of discontinuation of PIPAC in a multivariable logistic regression (Table 3).

The power of the multivariate analysis comparing the two groups was computed and determined to be 94%.

**Figure 1:**
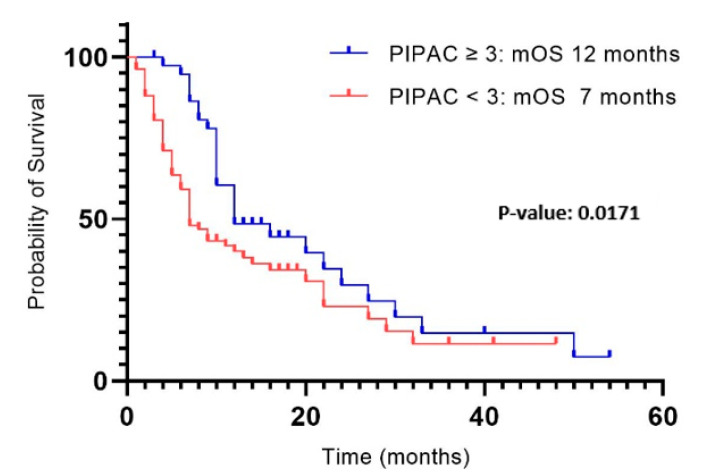
Survival from PIPAC1 in patients undergoing ST and 1–2 PIPAC.

The median follow-up period was 9 months, with a median overall survival from PIPAC1 of 10 months in the entire study population. We observed a significant difference in median overall survival between patients who underwent ST (12 months) and those who received only 1–2 PIPAC procedures (7 months) (*p*: 0.0171) (Figure 1).

Interestingly, the ST group exhibited a significant decrease in ascites (*p*: 0.04), while no efficacy was observed in the regression of other symptoms (Table 4).

Intraoperative complications occurred in two patients due to small bowel perforation during laparoscopic abdominal access. As for minor complications, CTCAE 1–2 adverse events were recorded in 47 procedures (13.98%). In detail, sixteen patients developed intense abdominal pain treated with analgesics. Twelve (3.57%) patients developed nausea or vomiting responsive to antiemetics. Ten (2.98%) patients developed a surgical site infection/dehiscence. Nine (2.68%) patients developed fever, and four of them were treated with antibiotics. Among the major complications, patients experienced CTCAE 3–4 adverse events in 13 procedures (3.86%). Four (1.19%) patients developed anemia and received a blood transfusion. Four (1.19%) patients had severe liver toxicity. Three (0.9%) patients developed neutropenia and two (0.6%) patients developed deep vein thrombosis. There were no severe complications needing reoperations or deaths during the in-hospital stay. The 30-day mortality rate was 1.78% (3 patients), while the 90-day mortality rate was 8.92% (15 patients).

Finally, a progressive and significant (*p*: 0.0404) increase in the number of patients receiving three PIPACs was documented between 2017 and 2023 (Table 5).

**Figure 2:**
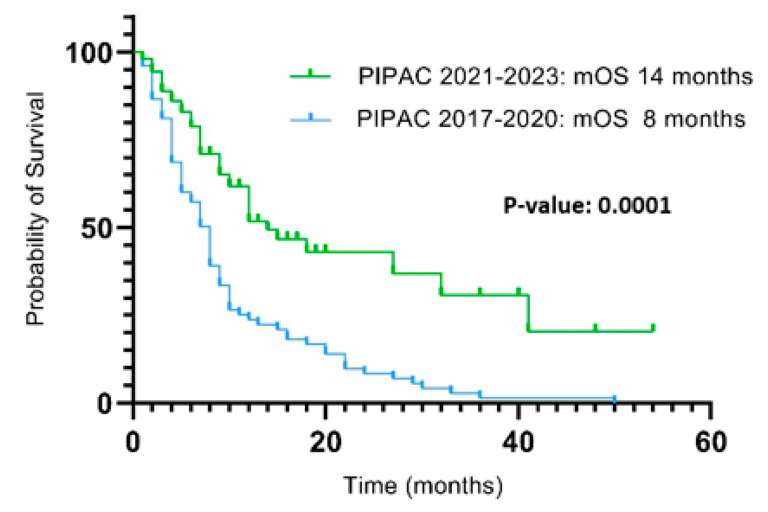
Survival from PIPAC1 between 2017 and 2020 and 2021 and 2023.

A significantly higher survival (*p*: 0.0001) was observed in patients treated up to 2020 compared to those treated in the following 3 years, with a mOS of 8 months (range 2–54) and 14 months (range 1–50), respectively (Figure 2).

## 4. Discussion

Pressurized Intraperitoneal Aerosol Chemotherapy (PIPAC), combined with systemic chemotherapy, represents an intraperitoneal therapeutic option to increase chemotherapeutic agents’ penetration into peritoneal metastases (PMs) [12,22,23]. According to the literature [24,25,26] and our findings, repeated treatment (at least three PIPACs) resulted in improved survival. A better survival rate with an increasing number of PIPAC procedures is also documented [6,27,28]. This provides a rationale for considering PIPAC treatment earlier in the treatment course, either alone or in combination with first-line systemic chemotherapy when tumor cells are more chemo-sensitive. Indeed, in patients with PM from pancreatic cancer, we initiated treatment already in the first metastatic line, with 33% of patients completing the entire treatment schedule. This percentage was higher compared to patients with PM from gastric and colorectal cancer, in whom PIPAC was performed in more advanced stages of the disease (27% and 26%, respectively).

The key finding of our analysis was that a small percentage (29%) of patients were able to complete at least 3 PIPAC procedures, resulting in survival benefits. Our experience reported less favorable results compared to the literature. According to a recent meta-analysis, 39% of patients complete the standard treatment protocol, with higher percentages in colon cancer (47%) and ovarian cancer (42%), and lower percentages in gastric cancer (34%), as well as hepatobiliary and pancreatic cancers (34%) [12]. A plausible explanation for these results may be the inclusion of patients treated in the early stages of our experience, where we observed a higher incidence of failures in completing the standard treatment compared to more recent patients. This finding is correlated with a progressive improvement in the surgical team’s experience and the systematic discussion of all patients eligible for PIPAC in multidisciplinary meetings. This, combined with an increasing understanding of the biological behavior of peritoneal surface malignancies (PSMs) and the therapeutic potential of PIPAC, has led to a progressive improvement in patient selection and, consequently, to an increase in the number of patients completing three PIPAC procedures over the years (Table 5), resulting in a concomitant improvement in oncological outcomes in terms of survival (Figure 2).

Although PIPAC is not a technically demanding surgical procedure, it presents significant challenges related to the learning curve of patient selection and optimal indication. Therefore, a well-defined multidisciplinary team is crucial to accelerating the learning curve, reducing untoward outcomes in the early period and improving oncological results. We reported a disease progression rate of 55.8% during treatment, which was the primary reason for treatment discontinuation. This finding, consistent with that reported by Ezanno (PD rate of 49.1%) [26], underscores the palliative nature of PIPAC treatment and emphasizes the importance of careful patient selection for this approach.

The most noteworthy reason for the discontinuation of PIPAC in our series was conversion to CRS during treatment. In our cohort, 10% of patients achieved a sufficient reduction in peritoneal disease burden to undergo CRS, prompting the discontinuation of PIPAC. Among them, nine patients had gastric peritoneal metastases, while the remaining patients had peritoneal metastases from colorectal cancer. The literature reports varying conversion rates to CRS and HIPEC [29], ranging from 7.6%, as reported by the meta-analysis of Di Giorgio et al. [12], to 26%, as reported by Casella et al. [4]. Considering the results of our previously published series [30] and the results of this study, the primary field of application for PIPAC as a neoadjuvant procedure appears to be gastric cancer with peritoneal metastases (GCPM). These results may open new therapeutic horizons, suggesting that PIPAC, which was originally a palliative treatment, might represent a neoadjuvant treatment option before curative surgery in selected patients with GCPM. In this context, the ongoing prospective, two-arm, randomized multicenter phase III clinical study PIPAC_VEROne is assessing the potential benefits of a more “intensive” bidirectional chemotherapy plus PIPAC treatment approach for patients with peritoneal-only oligometastatic disease [31].

Our findings regarding the potential predictors of early PIPAC discontinuation were consistent with those reported by Balmer et al. [32], indicating that ascites > 500 mL or symptoms of bowel obstruction at the first PIPAC procedure were associated with a higher risk of treatment discontinuation. However, high-volume ascites should not be considered a contraindication for PIPAC. Indeed, our results also demonstrated a significant reduction in ascites among patients undergoing systemic therapy (ST), which significantly improves patients’ quality of life and aligns with one of the therapeutic goals of PIPAC [33,34].

Notably, factors such as age, gender, BMI, or ECOG status at first PIPAC did not influence PIPAC discontinuation. This suggests that PIPAC can be repeatedly applied even in patients with a certain degree of functional impairment. However, it should be noted that patients with an ECOG performance status > 2 are excluded a priori from treatment.

The main limitation of this study is the small number of patients who underwent at least three PIPAC procedures with consequent numerical heterogeneity within the two comparison groups. Additionally, the wide time window of patient’s treatment has led to temporal heterogeneity among the analyzed cases. On the other hand, this study represents the real-life experience of a high-volume center that initiated this treatment in the early stages of the technique’s development.

## 5. Conclusions

PIPAC can offer a survival advantage after completing at least three treatments, de-spite the high rate of disease progression that results in a low completion rate among patients. Therefore, the identification of predictive factors such as the presence of ascites greater than 500 mL and bowel obstructive symptoms is essential to improve patient selection and, consequently, enhance oncological outcomes. However, due to its low complication rate, treatment safety, and potential neoadjuvant role, it remains a viable option for a carefully selected group of patients. The ongoing PIPAC cohort studies, shall shortly deliver more information on optimized selection criteria for PIPAC treatment.

## Figures and Tables

**Table 1:** Reasons for PIPAC interruption before PIPAC3.

Reasons for Interruption Before PIPAC3	*n* (%)
Disease Progression	67 (55.83%)
Patient’s Wish	13 (10.83%)
Conversion to CRS/HIPEC	12 (10%)
Other Medical Reasons	11 (9.16%)
Non-Access	9 (7.50%)
Death	4 (3.33%)
Negative Histology	2 (1.66%)
PIPAC complication	2 (1.66%)

CRS/HIPEC: Cytoreductive Surgery/Hyperthermic Intraperitoneal Chemotherapy. PIPAC: Pressurized Intraperitoneal Aerosol Chemotherapy.

**Table 2:** Potential predictors for interruption of standard treatment.

	PIPAC Procedures ≥ 3 *n* 48 (29%) Patients	PIPAC Procedures < 3 *n* 120 (71%) Patients	OR	CI for OR	*p*-Value
Age mean (SD)	63.12 (10.88)	60.27 (11.15)	1.024	CI 95% [0.991–1.058]	0.153
Gender male *n* (%)	21 (43.75%)	51 (42.50%)	1.076	CI 95% [0.531–2.181]	0.839
BMI mean (SD)	22.22 (4.16)	22.60 (4.02)	0.976	CI 95% [0.892–1.068]	0.599
ECOG ≥ 2 *n* (%)	9 (18.75%)	21 (17.50%)	1.159	CI 95% [0.474–2.864]	0.747
Primary Tumor *n* (%)					
Gastric Cancer	19 (39.58%)	50 (41.66%)	0.908	CI 95% [0.444–1.857]	0.791
Colorectal Cancer	8 (16.66%)	22 (18.33%)	1.069	CI 95% [0.424–2.699]	0.887
Hepato-bilio-pancreatic Cancer	19 (39.58%)	39 (32.50%)	1.483	CI 95% [0.722–3.045]	0.284
Other Primary Tumor	2 (4.16%)	9 (7.5%)	0.277	CI 95% [0.035–2.215]	0.226
Synchronous Carcinosis *n* (%)	20 (41.66%)	55 (45.83%)	1.213	CI 95% [0.597–2.465]	0.594
Median PCI (Min-Max) at PIPAC1	18 (3–36)	19 (2–39)	0.927	CI 95% [0.634–1.355]	0.696
Symptoms before PIPAC1 *n* (%)					
Ascites > 500 mL	10 (20.83%)	48 (40%)	0.366	CI 95% [0.157–0.853]	0.02
Bowel Obstruction	4 (8.33%)	29 (24.16%)	0.249	CI 95% [0.072–0.862]	0.028
Abdominal Pain	21 (43.75%)	72 (60%)	0.469	CI 95% [0.230–0.956]	0.037
Dysphagia	4 (8.33%)	14 (11.66%)	0.577	CI 95% [0.160–2.089]	0.403
Nausea	13 (27.08%)	27 (22.5%)	1.259	CI 95% [0.565–2.806]	0.574
Systemic Chemotherapy *n* (%)					
≥12 cycles	33 (68.75%)	66 (55%)	1.77	CI 95% [0.844–3.715]	0.131
≥3 lines	7 (14.58%)	26 (21.66%)	0.609	CI 95% [0.233–1.589]	0.311
Bimodal Treatment *n* (%)	33 (68.75%)	80 (66.66%)	1.101	CI 95% [0.521–2.330]	0.801

Statistical significance (*p* < 0.05): statistically significant values are underlined. BMI: body Mass Index. ECOG: Performance Status Scale. PCI: Peritoneal Cancer Index. PIPAC: Pressurized Intraperitoneal Aerosol Chemotherapy. SD: Standard Deviation.

**Table 3:** Multivariable logistic regression analysis of predictive factors.

Variable	OR	CI for OR	*p*-Value
Ascites > 500 mL	0.375	CI 95% [0.157–0.900]	0.028
Bowel Obstruction	0.264	CI 95% [0.74–0.947]	0.041
Abdominal Pain	0.671	CI 95% [0.315–1.429]	0.301

Statistical significance (*p* < 0.05): statistically significant values are underlined.

**Table 4:** Symptoms before PIPAC1 and after PIPAC3.

	Symptoms Before PIPAC1	Symptoms After PIPAC3	*p*-Value
Ascites *n* (%)	15 (36.59%)	4 (9.75%)	0.0429
Bowel Obstruction *n* (%)	3 (7.32%)	2 (4.87%)	0.644
Abdominal Pain *n* (%)	17 (41.46%)	15 (36.59%)	0.651
Dysphagia *n* (%)	3 (7.32%)	4 (9.75%)	0.692
Nausea *n* (%)	10 (24.39%)	9 (21.95%)	0.793

Statistical significance (*p* < 0.05): statistically significant values are underlined.

**Table 5:** Patients who underwent ST between 2017 and 2020 and 2021 and 2023.

Treatment Year	PIPAC ≥ 3 *n* (%)	PIPAC < 3 *n* (%)	*p*-Value
2017	1 (13%)	7 (87%)	0.0404
2018	4 (17%)	20 (83%)	
2019	8 (26%)	23 (74%)	
2020	5 (24%)	16 (76%)	
2021	11 (33%)	22 (67%)	
2022	14 (36%)	25 (64%)	
Mar-2023	5 (42%)	7 (58%)	

Statistical significance (*p* < 0.05): statistically significant values are underlined.

## Data Availability

The raw data supporting the conclusions of this article will be made available by the authors on request.
